# The Specific Binding and Promotion Effect of Azoles on Human Aldo-Keto Reductase 7A2

**DOI:** 10.3390/metabo13050601

**Published:** 2023-04-27

**Authors:** Wanying Wu, Tianqing Jiang, Haihui Lin, Chao Chen, Lingling Wang, Jikai Wen, Jun Wu, Yiqun Deng

**Affiliations:** 1 Guangdong Provincial Key Laboratory of Protein Function and Regulation in Agricultural Organisms, College of Life Sciences, South China Agricultural University, Guangzhou 510642, China; 2 Guangdong Laboratory for Lingnan Modern Agriculture, Guangzhou 510642, China; 3 Key Laboratory of Zoonosis of Ministry of Agriculture and Rural Affairs, South China Agricultural University, Guangzhou 510642, China

**Keywords:** azoles, AKR7A2, AFAR, docking, drug–protein interactions

## Abstract

Human AKR 7A2 broadly participates in the metabolism of a number of exogenous and endogenous compounds. Azoles are a class of clinically widely used antifungal drugs, which are usually metabolized by CYP 3A4, CYP2C19, and CYP1A1, etc. in vivo. The azole–protein interactions that human AKR7A2 participates in remain unreported. In this study, we investigated the effect of the representative azoles (miconazole, econazole, ketoconazole, fluconazole, itraconazole, voriconazole, and posaconazole) on the catalysis of human AKR7A2. The steady-state kinetics study showed that the catalytic efficiency of AKR7A2 enhanced in a dose-dependent manner in the presence of posaconazole, miconazole, fluconazole, and itraconazole, while it had no change in the presence of econazole, ketoconazole, and voriconazole. Biacore assays demonstrated that all seven azoles were able to specifically bind to AKR7A2, among which itraconazole, posaconazole, and voriconazole showed the strongest binding. Blind docking predicted that all azoles were apt to preferentially bind at the entrance of the substrate cavity of AKR7A2. Flexible docking showed that posaconazole, located at the region, can efficiently lower the binding energy of the substrate 2-CBA in the cavity compared to the case of no posaconazole. This study demonstrates that human AKR7A2 can interact with some azole drugs, and it also reveals that the enzyme activity can be regulated by some small molecules. These findings will enable a better understanding of azole–protein interactions.

## 1. Introduction

AKRs are a large protein superfamily that contains 16 subfamilies and has more than 190 members [1,2]. Presently, 15 human AKRs have been found and identified, among which AKR7A2 is deemed to participate in the metabolism of several compounds, e.g., reduction of SSA, acrolein, 2-CBA, and aflatoxin dialdehyde [1]. Human AKR7A3 is identified later than AKR7A2. Because human AKR7A2 and AKR7A3 can reduce the aflatoxin B_1_-dialdehyde to form nontoxic monoaldehyde or dialcohol, they are also called AFAR [3]. AKR7A2 has a high *K*_m_ for aflatoxin B_1_-dialdehyde [4], compared to which AKR7A3 has higher catalytic efficiency for this substrate [3]. AKR7A2 is mainly expressed in the brain and is thought to be an important SSA reductase [5] and likely to participate in the metabolism of GHB [6]. Furthermore, it has been reported that the AKR7A2 protein level is rising in the brains of Alzheimer’s disease patients [7]. In addition, AKR7A2 can also protect cells against aldehyde-induced cytotoxicity and genotoxicity, such as 4-hydroxynonenal [8,9] and methyl glyoxal [10], and it can metabolize anticancer drugs anthracycline doxorubicin and daunorubicin in cardiomyocytes [11]. These compounds or drugs mediate the transcription of AKR7A2 through Nrf2 [10,12] or NF-κB [13].

**Figure 1:**
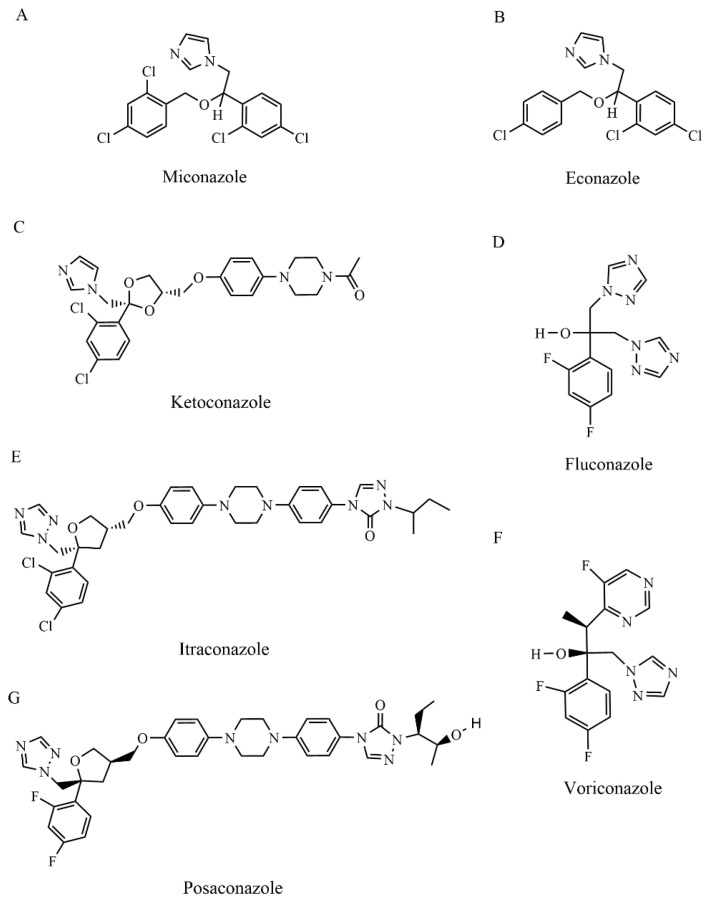
The molecular structures of azoles. (**A**) Miconazole, (**B**) Econazole, (**C**) Ketoconazole, (**D**) Fluconazole, (**E**) Itraconazole, (**F**) Voriconazole, (**G**) Posaconazole.

Azoles are a class of clinically widely used compounds for the treatment of various fungal infections [14]. To date, four generations and more than 40 azoles and drug candidates have been designed and developed [14]. Miconazole (Figure 1A) and econazole (Figure 1B) belong to the first-generation azole drugs, which were launched in 1960. The former is usually used to treat oropharyngeal or vulvovaginal candidiasis and other skin fungal infections [15], and the latter has poor water solubility and is generally used as cream and gel preparations for the treatment of tinea pedis and cruris etc. [16]. Ketoconazole (Figure 1C), fluconazole (Figure 1D), and itraconazole (Figure 1E) are the second-generation azole drugs, which change some drug structures to improve the safety and pharmacokinetics of the new drugs. Ketoconazole is the first oral and broad-spectrum antifungal drug [17], but it incurs a black box warning from US FDA because of the hepatotoxicity and pharmacokinetic interactions [18]. Fluconazole is the first used triazole drug [19], in which a triazole ring was introduced to replace the imidazole moiety in the azole structures, to enhance the selectivity for fungal P450 enzyme over mammalian P450 [20]. Therefore, fluconazole has a variety of advantages, including better specificity, greater water solubility, enhanced metabolic stability, as well as bioavailability, etc. [21,22,23]. Itraconazole was approved as an oral drug by the US FDA in 1992, and its structure is similar to that of ketoconazole, but it has a broader spectrum of antifungal activity than ketoconazole. Clinically, itraconazole is usually used for the treatment of endemic mycoses, *Candida albicans* infections, and invasive aspergillosis [24,25]. Remarkably, studies also have shown that itraconazole still has anticancer activity [26,27] and can inhibit enterovirus [28], influenza virus [29], and SARS-CoV-2 [30,31]. Voriconazole (Figure 1F) and posaconazole (Figure 1G) are the third-generation azole antifungal drugs, which have greater potency and safety profile, and more importantly, possess increased activity against the resistant types of fungus in comparison with the second-generation azole drugs, especially against *Aspergillus* spp. [32]. Voriconazole has wide-spectrum activity against a large number of fungi, such as *Aspergillus* spp., *Candida* spp., *Scedosporium*, *C. neoformans*, etc. [32]. It can be also used for the treatment of invasive aspergillosis for patients treated by hematopoietic stem cell transplant [33] and zygomycosis for immunocompromised patients [34]. Posaconazole is a new triazole antifungal drug that is structurally similar to itraconazole, which displays a broader spectrum of activity against a wide range of medically important fungal pathogens, including *Aspergillus* spp. [35,36], *Candida* spp. [37,38], *Cryptococcus* neoformans [39,40], and the zygomycetes [41]. Moreover, it is the first-line agent approved by the FDA for prophylaxis of IFD in patients with AML or MDS who are expected to develop prolonged neutropenia and at who are at a high risk of IFD, as well as stem cell transplant recipients [42]. Generally, posaconazole shows low hepatotoxicity and cardiotoxicity and has a safety profile for adults and children [43,44,45].

This study originated from an occasional observation in the experiment that pig aflatoxin-B_1_ aldehyde reductase exhibited an increased catalytic activity to the substrate aflatoxin dialdehyde in the presence of ketoconazole [46], which was used as the inhibitor of CYP3A and remained in the reaction system. Because azoles are widely used in humans, the study transfers to the interactions between azoles and human AKR7A2. The objective of this study was to further investigate the effect of some important and representative azoles, including posaconazole, voriconazole, itraconazole, fluconazole, ketoconazole, miconazole, and econazole, on the enzyme activity of human AKR7A2 and to provide a new perspective on the interactions between azoles and proteins.

## 2. Results

### 2.1. Effect of Azole Drugs on AKR7A2 Catalytic Kinetics

**Figure 2:**
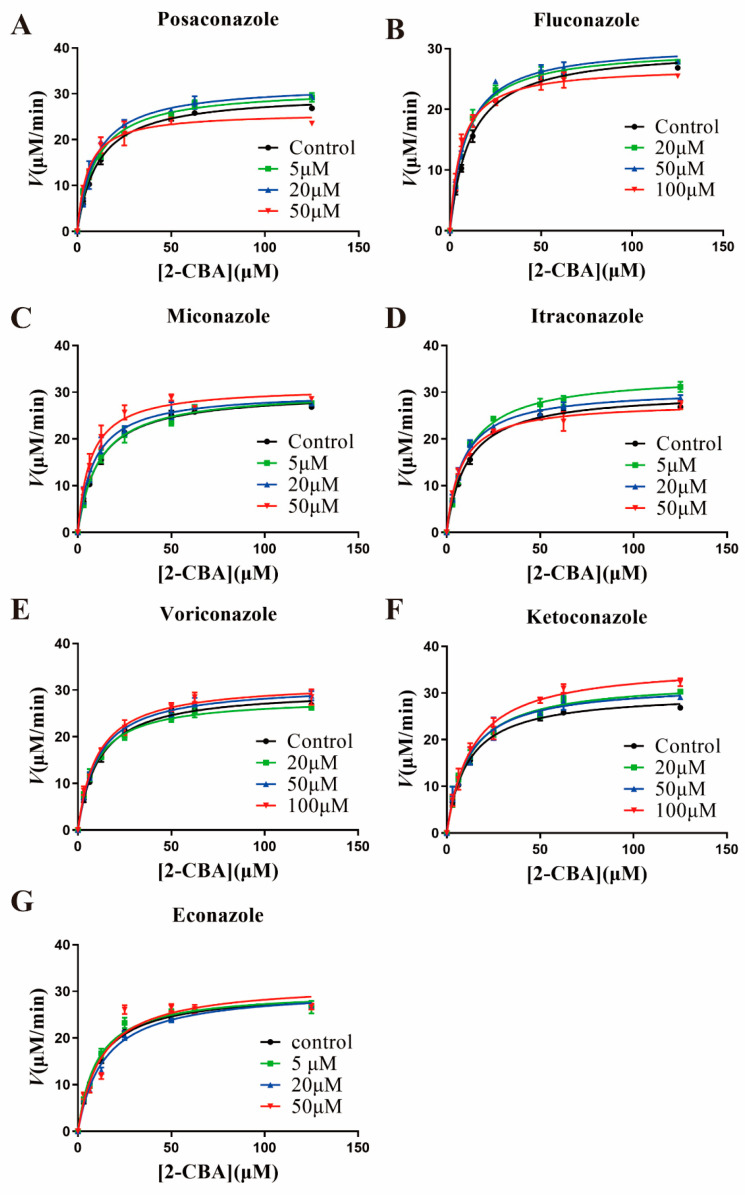
The Michaelis-Menten equation fittings of 2-CBA catalyzed by AKR7A2 in the absence or presence of azoles. (**A**). posaconazole, (**B**). fluconazole, (**C**). miconazole, (**D**). itraconazole, (**E**). voriconazole, (**F**). ketoconazole, (**G**). econazole. The steady-state kinetic measurements were repeated with at least three independent assays.

The steady-state kinetic parameters that AKR7A2 catalyzed 2-CBA were analyzed in the presence or absence of azole drugs. Generally, all the kinetic data conformed to the Michaelis-Menten equation, with R^2^ > 0.99 (Figure 2A–G). 

In the presence of posaconazole, miconazole, fluconazole, and itraconazole, the catalytic efficiency (*k*_cat_/*K*_m_) of AKR7A2 catalyzing 2-CBA enhanced significantly compared to that in the absence of these drugs, and this enhancement was in a dose-dependent manner (Table 1). However, the presence of voriconazole, ketoconazole, and econazole did not significantly change the catalytic efficiency of AKR7A2 (Table 1). Among the drugs examined, posaconazole showed the strongest promoting effect on AKR7A2. In contrast to that in the absence of posaconazole, *k*_cat_ had little variations in the presence of 5, 20, and 50 μM of the drug, which were 74.33 ± 1.27, 76.40 ± 1.74, and 61.75 ± 1.77 min^−1^ (Table 1), respectively. However, *K*_m_ presented an evident declining trend with the rising of posaconazole concentrations, which were 10.18 ± 0.66, 9.64 ± 0.84, and 5.56 ± 0.63 μM (Table 1), leading to an ascending trend of *k*_cat_/*K*_m_ on the whole and a maximum 74% increment. Interestingly, this phenomenon could be almost observed in the case of miconazole, fluconazole, and itraconazole. The *K*_m_ values corresponding to in the presence of 5, 20, and 50 μM miconazole are 11.34 ± 0.81, 8.73 ± 0.68, and 6.93 ± 0.70 μM, respectively, as well as 8.62 ± 0.50, 8.79 ± 0.54, and 6.12 ± 0.47 μM in the presence of 20, 50, and 100 μM fluconazole, as well as 10.91 ± 0.70, 8.84 ± 0.48, and 7.71 ± 0.63 μM in the presence of 5, 20, and 50 μM itraconazole. To some extent, these results can approximately reflect the enhancement of the enzyme’s affinity to the substrate. Although the dynamic change in *K*_m_ was not huge, it brought a significant elevation of *k*_cat_/*K*_m_, especially in the presence of the highest concentration of drugs, which have 67%, 64%, and 35% increments, respectively, compared with the absence of the drugs (Table 1). However, the regular decrease in *K*_m_ with elevation of the drug concentrations was not observed in the presence of voriconazole, ketoconazole, and econazole. The catalytic efficiency, *k*_cat_/*K*_m_, also did not have significant changes compared to without drugs. This means that the three drugs were not effective in AKR7A2 activity.

### 2.2. Affinity Determination of Azole Drugs with AKR7A2 by Biacore Assays

**Figure 3:**
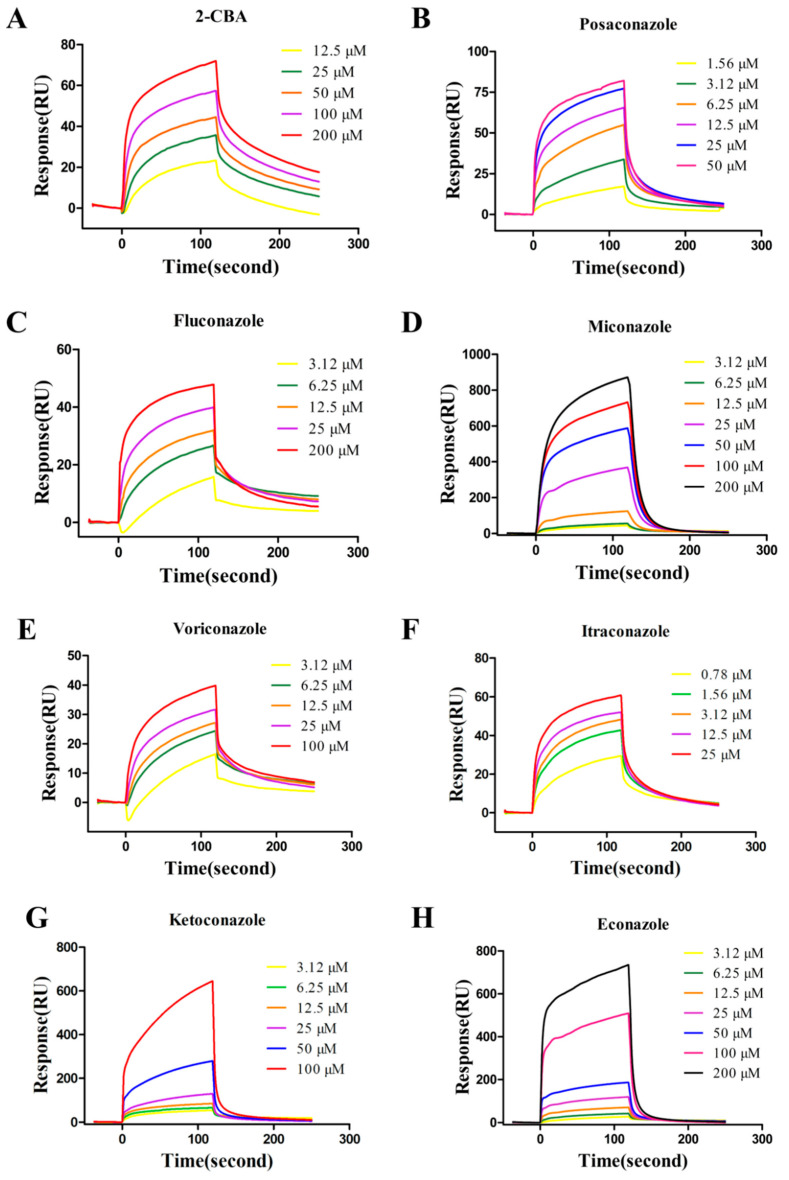
Representative binding sensorgrams for AKR7A2 with the compounds. The kinetic of AKR7A2 binding to 2-CBA (**A**), posaconazole (**B**), fluconazole (**C**), miconazole (**D**), voriconazole (**E**), itraconazole (**F**), ketoconazole (**G**), and econazole (**H**). The freshly purified AKR7A2 was covalently immobilized onto a CM5 sensor chip with ~20,000 RU. Different concentrations of compounds were injected at a flow rate of 30 µL/min. The data were fitted with a 1:1 binding model and gave the kinetic constants. All Biacore measurements were repeated with at least three independent experiments.

The kinetic interactions and affinity between the azole drugs and AKR7A2 were quantitatively investigated by Biacore assays. The typical binding sensorgrams of the drug molecules to the immobilized AKR7A2 were observed, which were all in a dose-dependent manner (Figure 3A–H). The association constant (*k*_a_) and dissociation constant (*k*_d_) were determined from the sensorgrams, and the *K*_D_ was calculated by *k*_d_/*k*_a_. The *K*_D_ for itraconazole was 0.81 ± 0.44 μM (Table 2), displaying the strongest binding affinity to AKR7A2 in all the examined azole drugs. The bindings of posaconazole, voriconazole, and fluconazole to AKR7A2 was slightly weaker than that of itraconazole, which were 1.35 ± 0.44, 2.98 ± 2.11, and 8.11 ± 4.83 μM, respectively (Table 2). Unexpectedly, the above four drugs’ affinity to AKR7A2 was all stronger than its model substrate 2-CBA (*K*_D_ = 19.30 ± 14.87 μM) (Table 2). Compared to posaconazole, voriconazole, and fluconazole, miconazole showed a weak binding, with a *K*_D_ of 84.94 ± 7.21 μM (Table 2). The binding of econazole to AKR7A2 was weaker than the drugs above, with a *K*_D_ of 565 ± 84 μM (Table 2). Ketoconazole had a huge *K*_D_ (1803 ± 438 μM) (Table 2), indicating the weakest binding to the protein in the examined drugs.

### 2.3. The Binding Area of Azole Drugs on AKR7A2 Predicted by Docking

**Figure 4:**
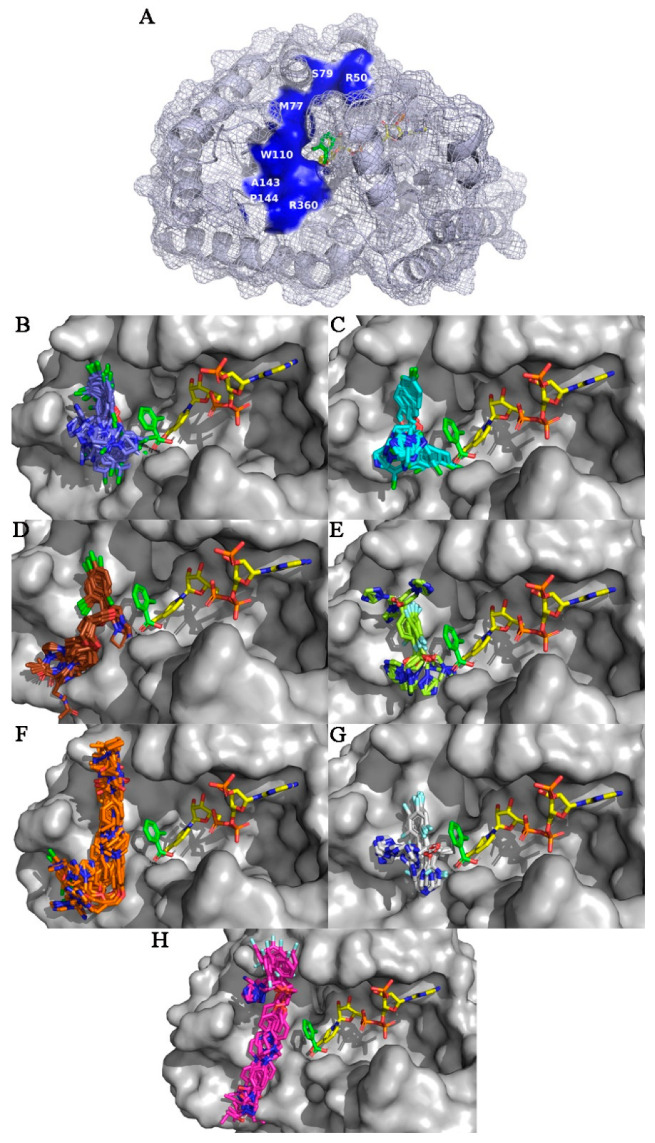
The binding area of the azoles on AKR7A2 predicted by blind docking. (**A**). The high-frequency binding area of azoles. Superposition of fourteen conformers of miconazole (**B**), nine conformers of econazole (**C**), eleven conformers of ketoconazole (**D**), nine conformers of fluconazole (**E**), eleven conformers of itraconazole (**F**), ten conformers of voriconazole (**G**), and seven conformers of posaconazole (**H**) at the entrance of substrate pocket. For the sake of clarity, residues 226–263 were not shown. Green: 2-CBA, yellow: NADPH. Color scheme: red for oxygen atoms, blue for nitrogen atoms, orange for phosphorus atoms, green for chlorine atoms, and light cyan for fluorine atoms. The figure was rendered with PyMOL [47].

To predict the potential binding sites of the azole drugs on AKR7A2, a blind docking study was performed without setting any binding regions beforehand. One hundred cycles of dockings were run, and the ligand conformers were clustered with an RMSD tolerance of 3.0 Å and ranked by the lowest docking energy for analysis. For all the azole drugs, the cluster analysis showed that their docking conformations were distributed dispersedly on the protein surface in the mass (Supplementary Materials Figures S1–S7). Interestingly, there existed a common high-frequency drug binding area located right in the middle of the α/β-barrel structure, consisting of Arg-50, Ser-79, Met-77, Trp-110, Ala-143, Pro-144, and Arg-360 from the top to the bottom (Figure 4A). Superposition of the docking conformer also displayed that the conformers with lower binding energy were close to the entrance of the substrate pocket, as shown in Figure 4B–H (corresponding to miconazole, econazole, fluconazole, ketoconazole, itraconazole, voriconazole, and posaconazole, respectively). This binding mode actually can restrict the movement of the substrate and stabilize its binding in the substrate pocket. Further analysis showed that when the azoles were in different conformations, they formed several hydrogen bonds with different residues in the substrate pocket. For miconazole and econazole, because their molecules are quite similar, they both can form hydrogen bonds with Trp-257 and Trp-260 (Supplementary Materials Figure S10A,B). For ketoconazole, it can potentially form hydrogen bonds with Asn-173, Thr-260, Arg-264, and Arg-360 (Supplementary Materials Figure S10C). For fluconazole, Arg-50, Asn-108, Asn-173, Ser-256, Thr-260, Arg-264, and Arg-360 participated in the formation of hydrogen bonds (Supplementary Materials Figure S10D). Itraconazole can form hydrogen bonds with Ser-256 and Arg-360 (Supplementary Materials Figure S10E). Voriconazole had hydrogen bond interactions with His-142, Thr-260, and Arg-360 (Supplementary Materials Figure S10F). Posaconazole can form hydrogen bonds with Trp-110, Ser-256, as well as with the atoms of the main chain (Supplementary Materials Figure S10G). It should be pointed out that, because the azoles contain benzene rings in their molecules, nonpolar interactions may also exist in the contacts between the azoles and some hydrophobic amino acids. The difference in binding affinity of the azoles with AKR7A2 was likely the result of a combination of polar and nonpolar interactions, and this process should be involved in the joint participation of multiple amino acid sites.

## 3. Discussion

Azoles exert their antifungal activity on the cell membranes by inhibiting the 14α-demethylation of lanosterol in the ergosterol biosynthetic pathway [48]. This process is implemented for most azoles by specific binding to the key enzyme of fungal ergosterol biosynthesis, CYP51 (lanosterol-14α-demethylase) [49,50]. There is also research showing that posaconazole can inhibit human cytomegalovirus replication by targeting human CYP51 [51]. In vivo, CYP 3A4, CYP2C19, CYP1A1, and flavin-containing monooxygenase 3 (FMO3) is thought to be responsible for the metabolism and clearance of some azoles, such as itraconazole and voriconazole [52,53]. Human AKR7A2 is an important enzyme involved in the metabolism of endogenous and exogenous compounds. To date, there is no report on the azole–protein interactions that human AKR7A2 participates in.

In terms of steady-state kinetics, this study first demonstrated that certain azoles were indeed able to promote the catalysis of AKR7A2 to a different extent. Because *K*_m_ can approximately reflect the affinity of the enzyme to the substrate, the decrease in *K*_m_ with the rising of the drug concentrations indicated that the drugs enhanced the binding of AKR7A2 with its substrate. Biacore assays directly demonstrated that the examined azoles indeed can specifically bind to AKR7A2. Interestingly, when comparing the two groups of results (Table 1 and Table 2), weaker bindings, such as ketoconazole (*K*_D_ = 1803 ± 438 μM) and econazole (*K*_D_ = 565 ± 84 μM), failed to promote the enzyme activity of AKR7A2, while other bindings of drugs with AKR7A2 did not exhibit the relation of affinity binding constants to the promotion effect, e.g., itraconazole had the strongest binding with *K*_D_ of 0.81 ± 0.44 μM and the weakest promotion effect. Voriconazole can bind to AKR7A2 with a high affinity (*K*_D_ = 2.98 ± 2.11 μM) while failing to enhance the enzyme activity. The affinity of miconazole to AKR7A2 (*K*_D_ = 84.94 ± 7.21 μM) was more than ten-fold lower than that of fluconazole (*K*_D_ = 8.11 ± 4.83 μM), whereas their promotion to AKR7A2 activity was on the same level. Only posaconazole showed a strong binding and maximum promotion to AKR7A2. It should be pointed out that, because of the poor solubility of azoles in water, the affinity of the substrate 2-CBA to the enzyme in the presence of drugs cannot be determined by Biacore assays. It needs other precise assays to further verify the hypothesis above.

The blind docking provided a structural explanation for the promotion of azoles to AKR7A2 enzyme activity. All the azoles were apt to bind at the entrance of the substrate pocket of AKR7A2, which indicates that the region is possibly a potential target of drugs. To all the azoles, this binding model seems to restrict the movement of 2-CBA in the substrate cavity and favorably enhances the specific binding of the substrate. However, this cannot explain why some azoles displayed the promotion to AKR7A2 catalysis. It is speculated that some azole molecules are possibly far away from 2-CBA, which failed to increase the binding of 2-CBA. Additionally, this should not rule out the possibility that other binding regions exist for some azoles.

**Figure 5:**
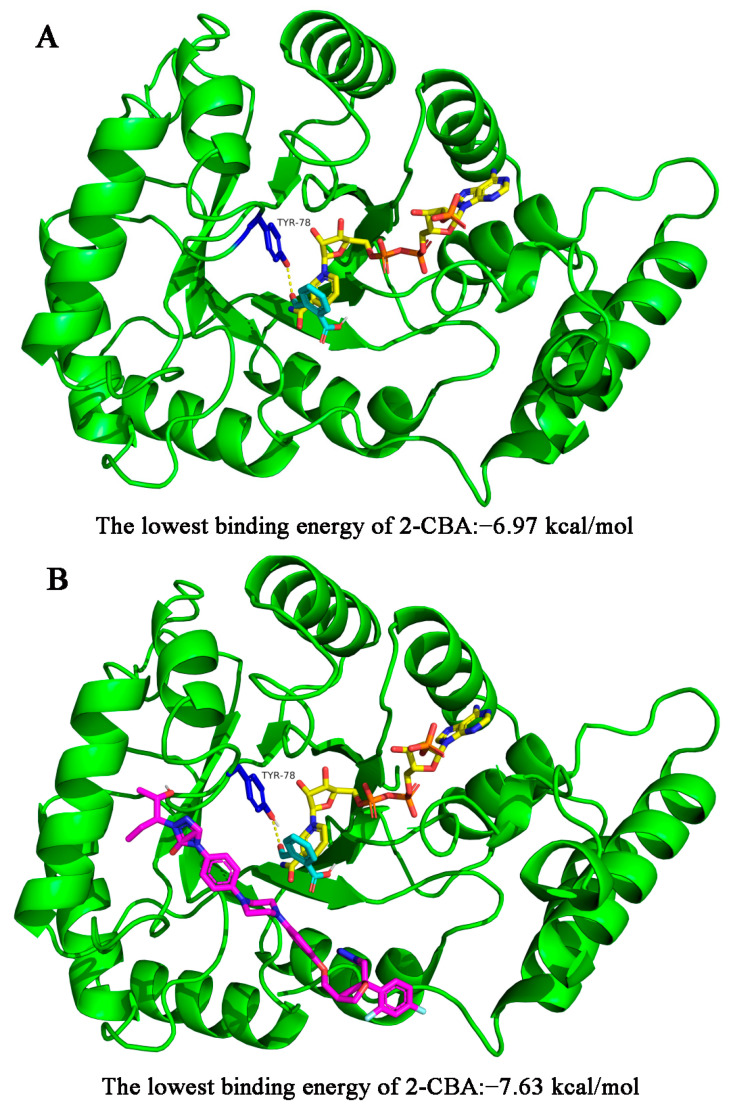
Comparison of the lowest binding energy of 2-CBA in the absence or presence of posaconazole. (**A**). The calculated lowest binding energy of 2-CBA was −6.97 kcal/mol in the absence of posaconazole. (**B**). The calculated lowest binding energy of 2-CBA was −7.63 kcal/mol in the presence of posaconazole. The simulations were performed with flexible docking by setting Tyr-78 as the flexible residue. Protein backbones are represented in green ribbon format. 2-CBA is depicted in cyan sticks, NADPH is depicted in yellow sticks, and posaconazole is depicted in purple sticks. The active center Tyr-78 residue is depicted in blue sticks. Color scheme: red for oxygen atoms, blue for nitrogen atoms, and orange for phosphorus atom. The figure was rendered with PyMOL [47].

To evaluate the effect of azoles on the catalysis of AKR7A2, we compared the alteration of the lowest binding energy in the absence or presence of posaconazole. Interestingly, under the condition of satisfying the premise that Tyr-87 functions as the electron donor, the lowest binding energy of 2-CBA in the absence of posaconazole was −6.97 kcal/mol (Figure 5A), while that in the presence of posaconazole decreased to −7.63 kcal/mol (Figure 5B). Further analysis showed that posaconazole could form hydrogen bonds with Asp-111, Lys-113, and Arg-264 in the substrate pocket (Supplementary Materials Figure S9). This result indicates that the presence of posaconazole can lower the binding energy of the substrate and stabilize its binding in the substrate cavity. It speculated that posaconazole located at the substrate pocket entrance can restrict the movement of 2-CBA, which is favorable for its catalysis. Moreover, because the posaconazole molecule contains several benzene rings, nonpolar interactions, such as π–π stacking and CH-π interaction, etc., also contribute to their binding.

**Scheme 1:**
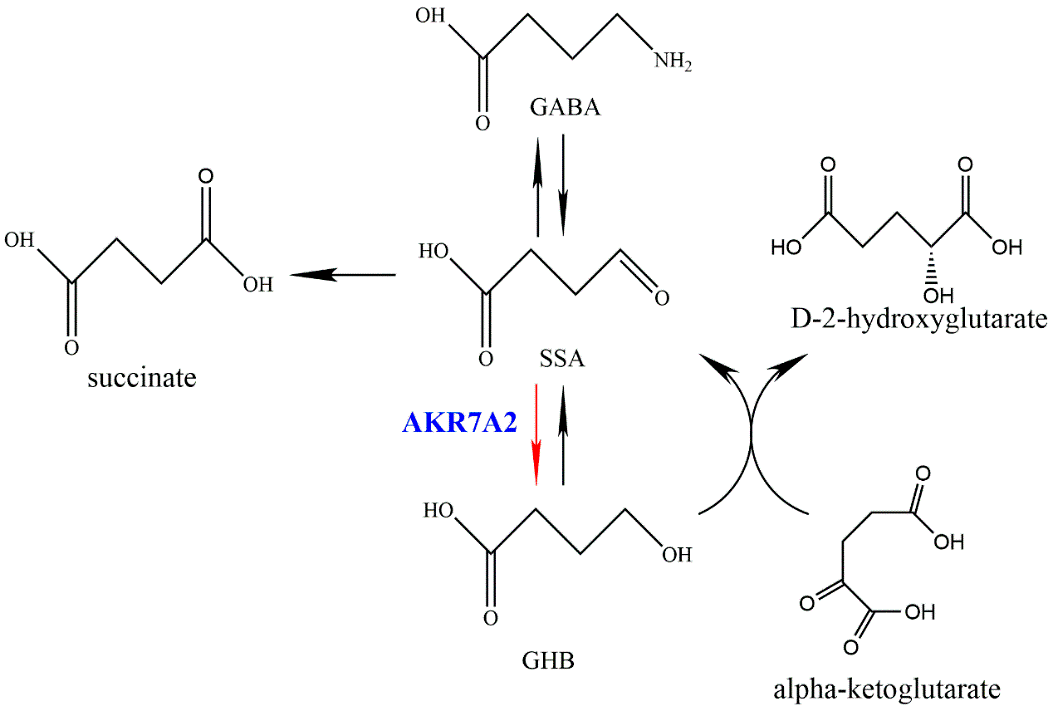
Pathway of SSA metabolism.

It should be noted that the azoles’ promotion to AKR7A2 enzyme activity is not just limited to a substrate 2-CBA. Concerning another classic substrate SSA of the AKR7 family, we also observed enhancement of AKR7A2 activity in the presence of some azoles, especially voriconazole, which may have more important physiological significance. Most azoles are large molecules (MW > 700 Da), and it is difficult for them to cross the BBB [54]. Voriconazole is the smallest triazole (MW: 349 Da), and it has been reported that cerebrospinal fluid concentrations of voriconazole after administration range from 0.08–3.93 µg/mL in patients [55], and voriconazole concentrations in the brain tissue specimens of two dead patients reach up to 11.8 µg/mg and 58.5 µg/mg, indicating that voriconazole can penetrate through the BBB and penetrate the brain tissue. In this study, the steady-state kinetics with SSA as a substrate displayed that voriconazole was able to efficiently promote the catalysis of AKR7A2 in the concentration range of 0–50 µM (Supplementary Materials Figure S8 and Table S1). SSA is an important intermediate and undergoes complex metabolism in brains (Scheme 1). It is supposed that the presence of voriconazole may change the relative balance of GABA, SSA, and GHB, promoting AKR7A2 to produce more GHB (Scheme 1), which needs in vivo assays to further validate.

In summary, this study reported the discovery that azoles, including miconazole, econazole, fluconazole, ketoconazole, itraconazole, voriconazole, and posaconazole, which can specifically bind to human AKR7A2, among which posaconazole, miconazole, fluconazole, and itraconazole had the capacity to promote AKR7A2 enzyme activity in a dose-dependent manner. A docking study showed that there probably existed a common drug-binding region at the entrance of the substrate cavity. From the point of view of binding energy, the binding of posaconazole was favorable to the stability of 2-CBA in the catalytic center. This study reveals that some azoles may have a potential and undiscovered protein target, which contributes to understanding the new pharmaceutical effects of azoles and promoting the application of the drugs. Meanwhile, the study demonstrates that human AKR7A2 activity can be regulated by certain small molecules. In addition, our study provides evidence that azoles can promote human AKR7A2 activity in vitro. It needs more in vivo research to further investigate the interactions of azole-AKR7A2.

## 4. Materials and Methods

### 4.1. Materials

2-CBA was purchased from Sigma-Aldrich (St. Louis, MO, USA). Isopropyl-β-D-thiogalactoside (IPTG) and NADPH, produced by Gen-View Scientific Inc (Wellington, FL, USA), were purchased from Beijing Dingguo Changsheng Biotechnology Limited Corporation. Kanamycin sulfate and BCA protein quantitation kits were also from Beijing Dingguo Biotechnology Corporation. A 5-mL HisTrap HP column was purchased from GE Healthcare (Uppsala, Sweden). High-fidelity DNA polymerase KOD FX was purchased from Toyobo (Osaka, Japan). T4 DNA ligase was purchased from New England Biolabs (Northampton, MA, USA). All primers were synthesized in Sangon Biotech (Guangzhou, China). All the other chemicals used were of analytical grade.

### 4.2. Cloning, Protein Expression, and Purification of Human AKR7A2

Human *akr7A2* gene sequence (NM_001320979.1) was amplified from human HEK293T cells and then was introduced to the homology arm of the expression vector at both ends of the sequence (underlined) as follows: the forward primer 5′-aagaaggagatatacATGCATCATCACCATCACCACCTGAGTGCCGCG-3′ and the reverse primer 5′-tgtcgacggagctcgCTAGCGGAAGTAGTTGGGACATTC-3′. The PCR reaction was performed using a thermos-cycling program that ran for 3 min at 94 °C 3 min and then proceeded for 34 cycles at 98 °C for 10 s, 52 °C for 30 s, 68 °C for 90 s, and a final extension step at 68 °C for 5 min. The PCR product was ligated into the pET-28a expression vector (Novagen) by homologous recombination. The final sequence was confirmed by DNA sequencing. The recombinant AKR7A2 was expressed in *E.coli* BL21 (DE3) pLysS cells. A single colony containing the recombinant AKR7A2 expression plasmid was inoculated into 5 mL of Luria Broth (LB) with 50 μg/mL kanamycin and incubated for 5 h at 37 °C and 150 rpm. Then, the bacteria solution was transferred into 500 mL of LB to cultivate until the solution OD_600_ reached 0.6~0.8. At this time, AKR7A2 expression was induced by the addition of 1 mM IPTG at 16 °C for 24 h. The cells were harvested by centrifugation with 3500× *g* for 30 min at 4 °C. The cell pellets were resuspended in ice-cold ~40 mL of buffer A (100 mM sodium phosphate, 20 mM imidazole, and 500 mM sodium chloride, pH 7.4). The cell suspension was gently stirred for 1 h at 4 °C and sonicated over ice (15% power, working for 10 s, resting for 10 s, and a total of 30 min). The lysate was centrifuged at 6500 rpm for 30 min at 4 °C, and the supernatant was filtered through a 0.45 μm nylon membrane before being loaded onto a 5 mL HisTrap HP column pre-equilibrated with buffer A. The recombinant proteins were eluted with a gradient of 150–500 mM imidazole in buffer B (100 mM sodium phosphate, 500 mM sodium chloride, 500 mM imidazole, pH 7.4). The protein purity exceeded 99%, as judged by SDS-PAGE and Coomassie brilliant blue staining. The fractions containing the protein of interest were combined and dialyzed against a buffer containing 100 mM sodium phosphate (pH 7.4), and 500 mM NaCl at 4 °C for 24 h. The total protein concentration was determined by the BCA method. Aliquots of proteins were stored at −80 °C until being used.

### 4.3. Steady-State Kinetics

The steady-state kinetic parameters of AKR7A2 catalyzing 2-CBA were measured on a UV-2550 spectrophotometer (SHIMADZU, Japan) in the absence or presence of azole drugs. All drugs were dissolved with DMSO. Fluconazole, voriconazole, and ketoconazole were all made up of 2, 5, and 10 mM of mother solution. Because of the small solubility in DMSO, posaconazole, miconazole, itraconazole, and econazole were all made up of 0.5, 2 and 5 mM of mother solution. The steady-state kinetic assays were performed, as before [46], with some modifications. Briefly, the reaction mixtures consisted of 100 mM sodium phosphate buffer (pH 7.4), 0.2 mM NADPH, 0.4 μM purified recombinant protein AKR7A2 and different concentrations of the substrate 2-CBA (3.13–250 μM) to a final volume of 800 μL. The enzymatic activity of AKR7A2 was not inhibited by 1% of DMSO. The reactions were initiated by the addition of 2-CBA and were monitored for 4 min at 25 °C. Using GraphPad Prism 8.0, data from at least three separate experiments were fitted to the Michaelis-Menten equation to obtain the kinetic parameters (*K*_m_, *k*_cat_).

### 4.4. Biacore Assays

All experiments were performed on a Biacore 8k instrument (GE, Healthcare). According to the manufacturer’s handbook, about 1 mg/mL of freshly purified AKR7A2 protein was diluted with 10 mM sodium acetate buffer (pH 5.0) and was immobilized on a sensor chip CM5, via amine coupling, to a level of ~20,000 resonance units (RU). A reference surface was prepared in parallel with the addition of the buffer and was used for the subtraction of nonspecific binding. The analytes 2-CBA and azole drugs were diluted in a running buffer containing 2% DMSO. The kinetic constants of the compounds were determined with multi-cycle kinetics with at least five consecutive injections with an increasing compound concentration ranging from 0–200 μM, depending on the drug solubility. All samples were injected at a flow rate of 30 μL/min. The experimental sensorgrams were analyzed by Biacore 8k Evaluation Software Version 1.1.1.7442. The resulting curves were fitted with a 1:1 binding model, which gave the kinetic constants (*k*_a_, *k*_d_, and *K*_D_). All measurements were repeated in at least three independent experiments.

### 4.5. Docking Studies

A blind docking study was performed for predicting the binding sites of azole drugs in AKR7A2 using the program AutoDock 4.2. The human AKR7A2 crystal structure (PDB ID: 2BP1) available was used as the protein receptor. The molecular structures of all azole drugs were drawn by ChemOffice 2017 (PerkinElmer Informatics, Inc., Waltham, MA, USA). Before docking, the energy of protein and drug molecules was minimized. To accommodate the whole protein molecule, the grid box was set to 142 × 126 × 126 points with the center at −17.107, 26.624, −1.453, and spacing of 0.375 Å. The Lamarckian genetic algorithm was used to determine the globally optimized conformations of the ligand with the following settings: Number of GA Runs: 100; Population Size: 150; Maximum number of evaluations: 2,500,000; Maximum number of generations: 27,000; and, the other parameters were set to their default values. The docking conformations were clustered with an RMS deviation (RMSD) of 3.0 Å, and the reasonable conformers were analyzed and plotted by PyMOL [47].

Based on the blind docking result of posaconazole, a flexible docking study was performed in the presence or absence of the posaconazole molecule. Tyr-78 was set as the flexible residue. Posaconazole conformer with the lowest binding energy in blind docking was extracted and integrated into the protein molecule. This protein complex containing posaconazole was used as the docking receptor. The grid size was set to be 60 × 60 × 60 with the center at −18.16, 29.26, 4.894 and spacing of 0.375 Å. The Lamarckian genetic algorithm was still applied to search for the optimal ligand conformation and orientation within the active pocket. The detailed docking parameters used were as described previously. The docking conformations were clustered into groups with RMSD of 1.0 Å.

### 4.6. Data Analysis

The kinetic data were fitted to the Michaelis-Menten equation [56,57] using GraphPad Prism 8.0 (GraphPad Software, San Diego, CA, USA). The kinetic parameters *K*_m_ and *k*_cat_ were generated by the fitting. The resulting data were expressed as the mean ± standard deviation (SD) based on at least three independent experiments. Statistical significance was analyzed by ANOVA, and the significance was defined as * *p* < 0.05, ** *p* < 0.01, *** *p* < 0.001, or **** *p* < 0.0001.

## Figures and Tables

**Table 1:** Kinetic parameters of effect of azole on AKR7A2 enzyme activity.

Protein/Drug	Drug Concentration (μM)	*K*_m_ (μM)	*k*_cat_ (min^−1^)	*k*_cat_/*K*_m_ (min^−1^ ∗ μM^−1^)	Normalized Value (*k*_cat_/*K*_m_)
AKR7A2	0	11.21 ± 0.53	71.72 ± 0.93	6.40	1.00
posaconazole	5	10.18 ± 0.66	74.33 ± 1.27	7.30	1.14
posaconazole	20	9.64 ± 0.84	76.40 ± 1.74	7.93 *	1.24
posaconazole	50	5.56 ± 0.63	61.75 ± 1.77	11.10 ****	1.74
miconazole	5	11.34 ± 0.81	72.31 ± 1.37	6.38	1.00
miconazole	20	8.73 ± 0.68	71.78 ± 1.41	8.22	1.28
miconazole	50	6.93 ± 0.70	74.13 ± 1.75	10.69 **	1.67
fluconazole	20	8.62 ± 0.50	71.68 ± 1.04	8.31	1.30
fluconazole	50	8.79 ± 0.54	73.24 ± 1.15	8.33	1.30
fluconazole	100	6.12 ± 0.47	64.35 ± 1.11	10.51 ***	1.64
itraconazole	5	10.91 ± 0.70	80.53 ± 1.42	7.38	1.15
itraconazole	20	8.84 ± 0.48	73.12 ± 1.00	8.27 **	1.29
itraconazole	50	7.71 ± 0.63	66.42 ± 1.38	8.61 ***	1.35
voriconazole	20	9.33 ± 0.52	67.56 ± 0.98	7.24	1.13
voriconazole	50	10.77 ± 0.56	74.24 ± 1.01	6.89	1.08
voriconazole	100	10.35 ± 0.77	75.51 ± 1.49	7.30	1.14
ketoconazole	20	11.95 ± 0.90	78.09 ± 1.61	6.53	1.02
ketoconazole	50	11.13 ± 0.98	76.28 ± 1.82	6.85	1.07
ketoconazole	100	13.47 ± 1.13	86.37 ± 2.09	6.41	1.00
econazole	5	10.28 ± 0.90	71.64 ± 1.71	6.97	1.09
econazole	20	13.96 ± 1.02	72.76 ± 1.55	5.21	0.81
econazole	50	12.73 ± 2.26	75.70 ± 3.81	5.95	0.93

* *p* < 0.05, ** *p* < 0.01, *** *p* < 0.001, **** *p* < 0.0001. The data represent the mean ± SD of at least three replicates and were analyzed by GraphPad Prism 8.0.

**Table 2:** Binding kinetic parameters of AKR7A2 with 2-CBA and drug molecules determined by Biacore.

Analytes	M.W. (Da)	*k*_a_ (M^−1^s^−1^) × 10^−3^	*k*_d_ (s^−1^) × 10^2^	*K*_D_ (μM)
2-CBA	150.13	1.19 ± 1.36	2.29 ± 2.02	19.30 ± 14.87
posaconazole	700.78	17.3 ± 16.7	2.98 ± 0.59	1.35 ± 0.44
itraconazole	705.64	32.4 ± 8.0	2.63 ± 0.35	0.81 ± 0.44
voriconazole	349.32	5.64 ± 3.41	1.68 ± 0.72	2.98 ± 2.11
fluconazole	306.28	2.68 ± 0.95	2.18 ± 0.46	8.11 ± 4.83
miconazole	416.13	0.63 ± 0.23	5.36 ± 0.17	84.94 ± 7.21
ketoconazole	531.43	0.070 ± 0.044	12.6 ± 1.91	1803 ± 438
econazole	381.68	0.28 ± 0.086	15.9 ± 0.72	565 ± 84

The binding constants of AKR7A2/analyte interactions were determined at 25 °C, and each experiment was repeated at least three times.

## Data Availability

The data from this study and data repository access are available upon a reasonable request addressed to the corresponding author. Data is not publicly available due to privacy or ethical restrictions.

## Supplementary Materials

The following supporting information can be downloaded at: https://www.mdpi.com/article/10.3390/metabo13050601/s1, Figure S1. The cluster distribution of econazole docking into AKR7A2; Figure S2. The cluster distribution of fluconazole docking into AKR7A2; Figure S3. The cluster distribution of itraconazole docking into AKR7A2.; Figure S4. The cluster distribution of ketoconazole docking into AKR7A2; Figure S5. The cluster distribution of miconazole docking into AKR7A2; Figure S6. The cluster distribution of posaconazole docking into AKR7A2; Figure S7. The cluster distribution of voriconazole docking into AKR7A2; Figure S8. The Michaelis-Menten equation fittings of SSA catalyzed by AKR7A2 in the absence or presence of voriconazole; Figure S9 The interactions of posaconazole with the residues in the substrate pocket; Figure S10 The hydrogen bonds between the azoles and the residues in AKR7A2; Table S1. Kinetic parameters of effect of voriconazole on AKR7A2 enzyme activity with SSA as a substrate.

## Abbreviations

AKR, aldo-keto reductase; SSA, succinic semialdehyde; 2-CBA, 2-carboxybenzaldehyde; GHB, γ-hydroxybutyrate; AFAR, aflatoxin aldehyde reductase; CYP, Cytochrome P450; IPTG, isopropyl-β-D-thiogalactoside; Nrf2, nuclear factor erythroid 2-related factor 2; FDA, Food and Drug Administration; IFD, invasive fungal diseases; AML, acute myeloid leukemia; MDS, myelodysplastic syndromes; BBB, blood-brain barrier.
