# Supplementary material for: The Specific Binding and Promotion Effect of Azoles on Human Aldo-Keto Reductase 7A2
Source: Metabolites. 2023 Apr 27;13(5):601. doi: 10.3390/metabo13050601 (PMC10221418; doi:10.3390/metabo13050601)
Supplement: Supplementary file 1 [file metabolites-13-00601-s001.zip › metabolites-2303402-supplementary.pdf]

# Supplementary data

# The specific binding and promotion effect of azoles to human aldo-keto reductase 7A2

Wanying Wu[a,b,c,1], Tianqing Jiang[a,b,c,1], Haihui Lin[a,b,c], Chao Chen[a,b,c], Lingling Wang[a,b,c], Jikai Wen[a,b,c], Jun Wu[a,b,c,*], Yiqun Deng[a,b,c,*]

[a] Guangdong Provincial Key Laboratory of Protein Function and Regulation in Agricultural Organisms, College of Life Sciences, South China Agricultural University, Guangzhou 510642, Guangdong, China

[b] Guangdong Laboratory for Lingnan Modern Agriculture, Guangzhou 510642, Guangdong, China

[c] Key Laboratory of Zoonosis of Ministry of Agriculture and Rural Affairs, South China Agricultural University, Guangzhou 510642, Guangdong, China

[1]These authors contributed equally to this work.

[*] Corresponding author, e-mail: yqdeng@scau.edu.cn, wujun@scau.edu.cn; Tel./Fax: +86-20-3860-4967

**Contents:**

**Figure S1.** The cluster distribution of econazole docking into AKR7A2.

**Figure S2.** The cluster distribution of fluconazole docking into AKR7A2.

**Figure S3.** The cluster distribution of itraconazole docking into AKR7A2.

**Figure S4.** The cluster distribution of ketoconazole docking into AKR7A2.

**Figure S5.** The cluster distribution of miconazole docking into AKR7A2.

**Figure S6.** The cluster distribution of posaconazole docking into AKR7A2.

**Figure S7.** The cluster distribution of voriconazole docking into AKR7A2.

**Figure S8.** The Michaelis-Menten equation fittings of SSA catalyzed by AKR7A2 in the absence or presence of voriconazole.

**Figure S9** The interactions of posaconazole with the residues in the substrate pocket.

**Figure S10** The hydrogen bonds between the azoles and the residues in AKR7A2.

**Table S1.** Kinetic parameters of effect of voriconazole on AKR7A2 enzyme activity with SSA as a substrate

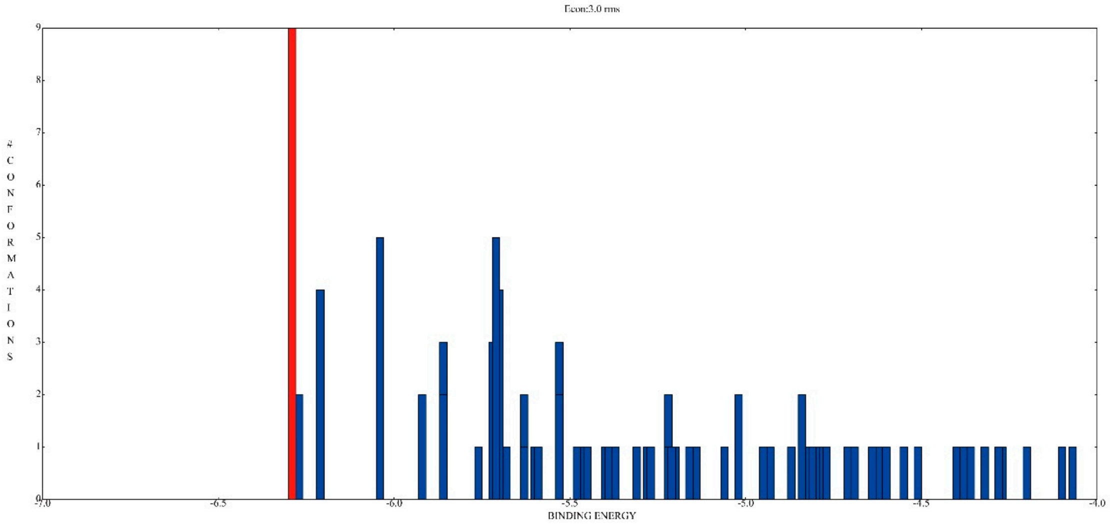


**Figure S1** The cluster distribution of econazole docking into AKR7A2.

The red column represents the conformers binding at the entrance of the substrate pocket

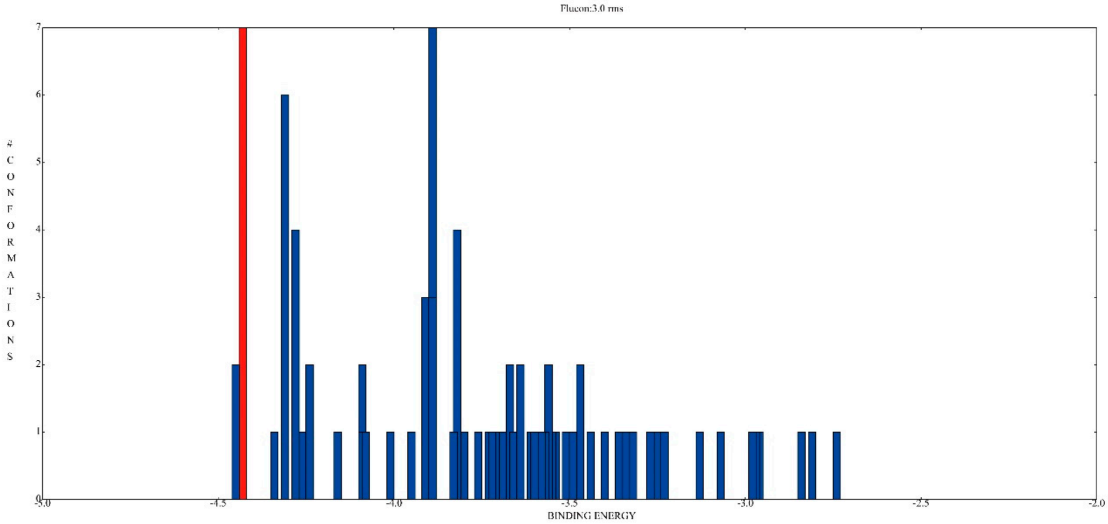


**Figure S2** The cluster distribution of fluconazole docking into AKR7A2.

The red column represents some conformers binding at the entrance of the substrate pocket

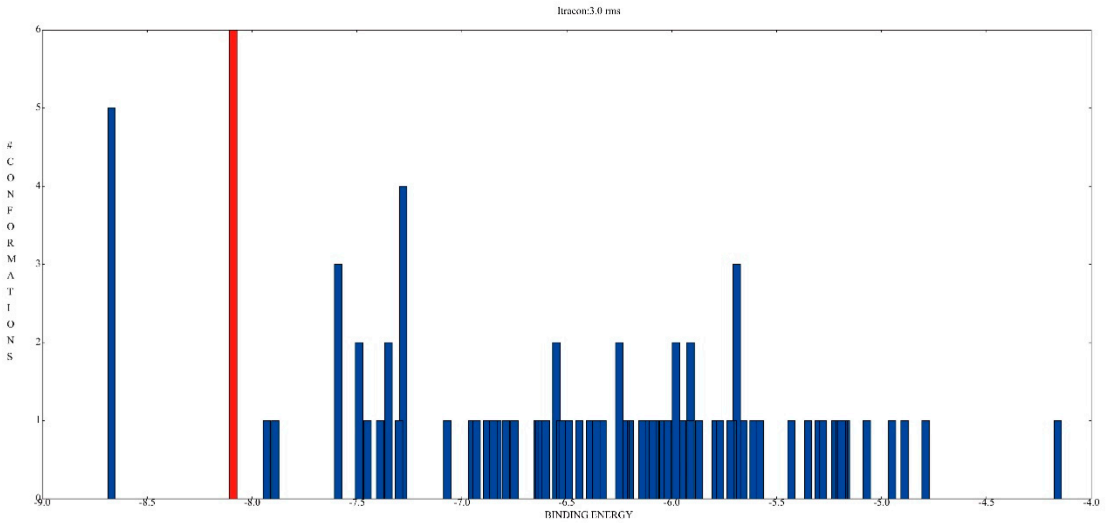


**Figure S3** The cluster distribution of itraconazole docking into AKR7A2.

The red column represents some conformers binding at the entrance of the substrate pocket

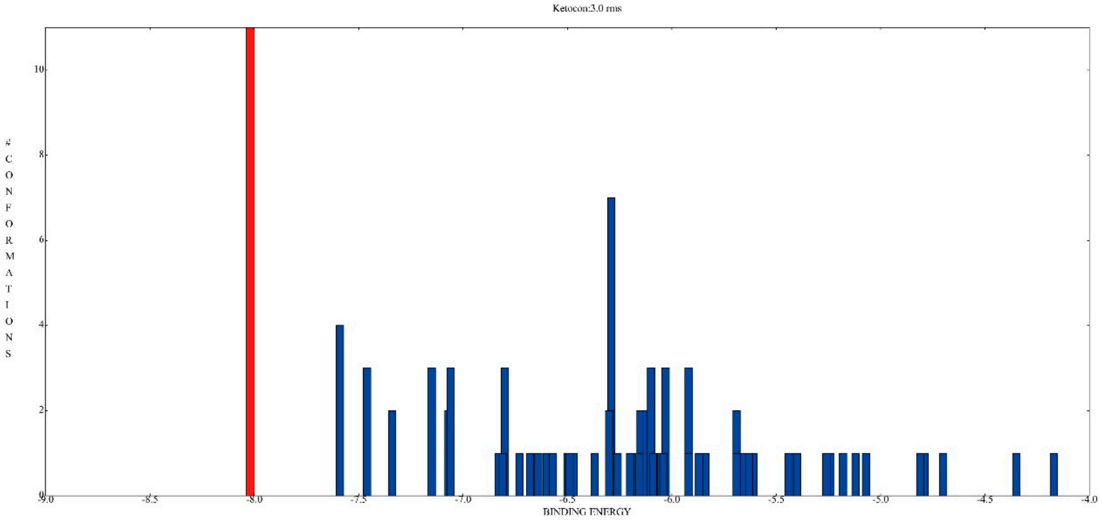


**Figure S4** The cluster distribution of ketoconazole docking into AKR7A2.

The red column represents the conformers binding at the entrance of the substrate pocket

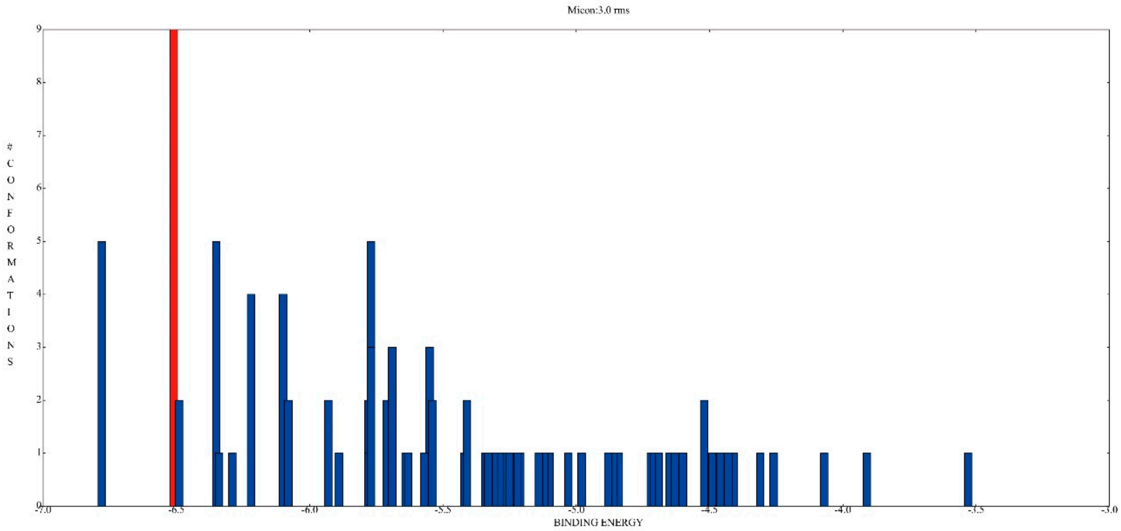


**Figure S5** The cluster distribution of miconazole docking into AKR7A2.

The red column represents some conformers binding at the entrance of the substrate pocket

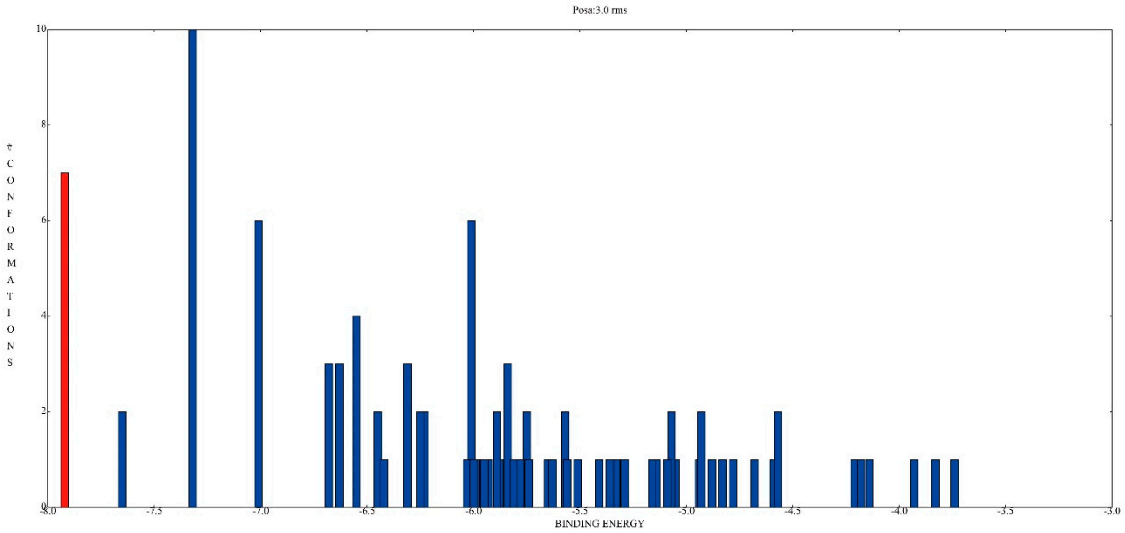


**Figure S6** The cluster distribution of posaconazole docking into AKR7A2.

The red column represents the conformers binding at the entrance of the substrate pocket

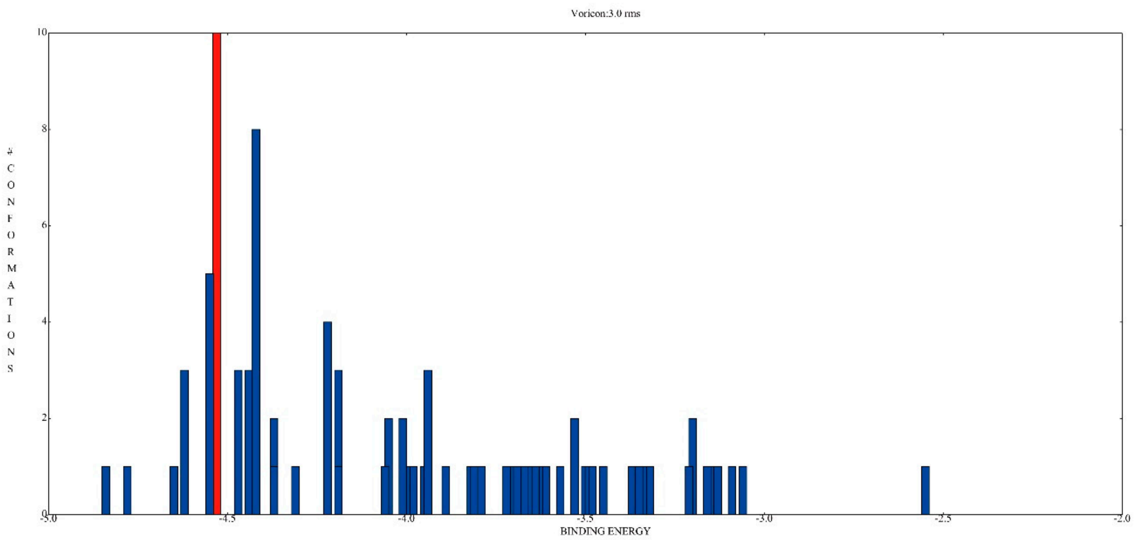


**Figure S7** The cluster distribution of voriconazole docking into AKR7A. The red column represents the conformers binding at the entrance of the substrate pocket

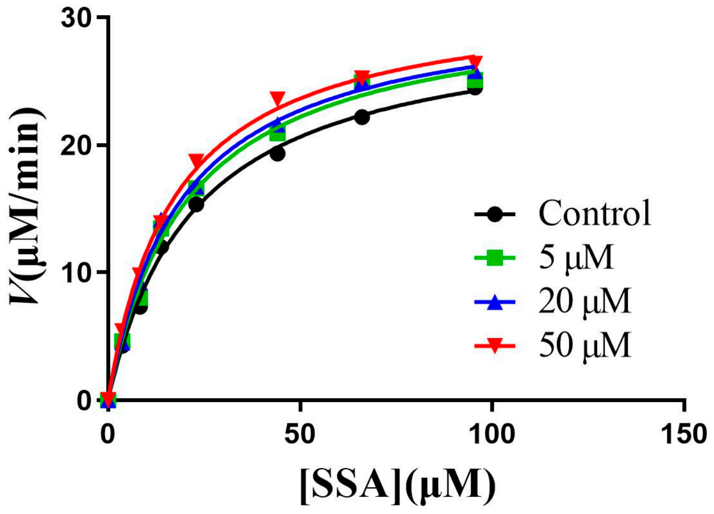


**Figure S8** The Michaelis-Menten equation fittings of SSA catalyzed by AKR7A2 in the absence or presence of voriconazole

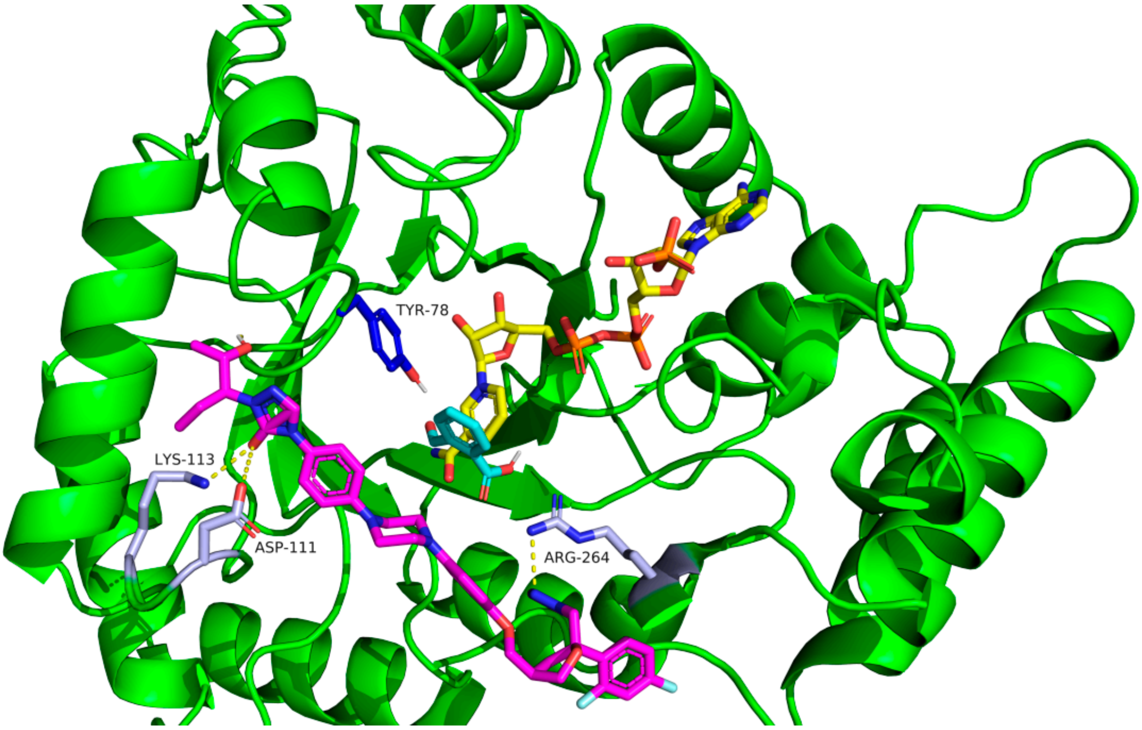


**Figure S9** The interactions of posaconazole with the residues in the substrate pocket. The simulations were performed with flexible docking by setting Tyr-78 as the flexible residue. Protein backbones are represented as green ribbon format. 2-CBA is depicted in cyan sticks, NADPH is depicted in yellow sticks, and posaconazole is depicted in purple sticks. The active center Tyr-78 residue is depicted in blue sticks. Color scheme: red for oxygen atoms, blue for nitrogen atoms, and orange for phosphorus atom. Hydrogen bonds are shown as yellow dashed lines. Asp-111, Lys-113, and Arg-264 can form hydrogen bonds with posaconazole.

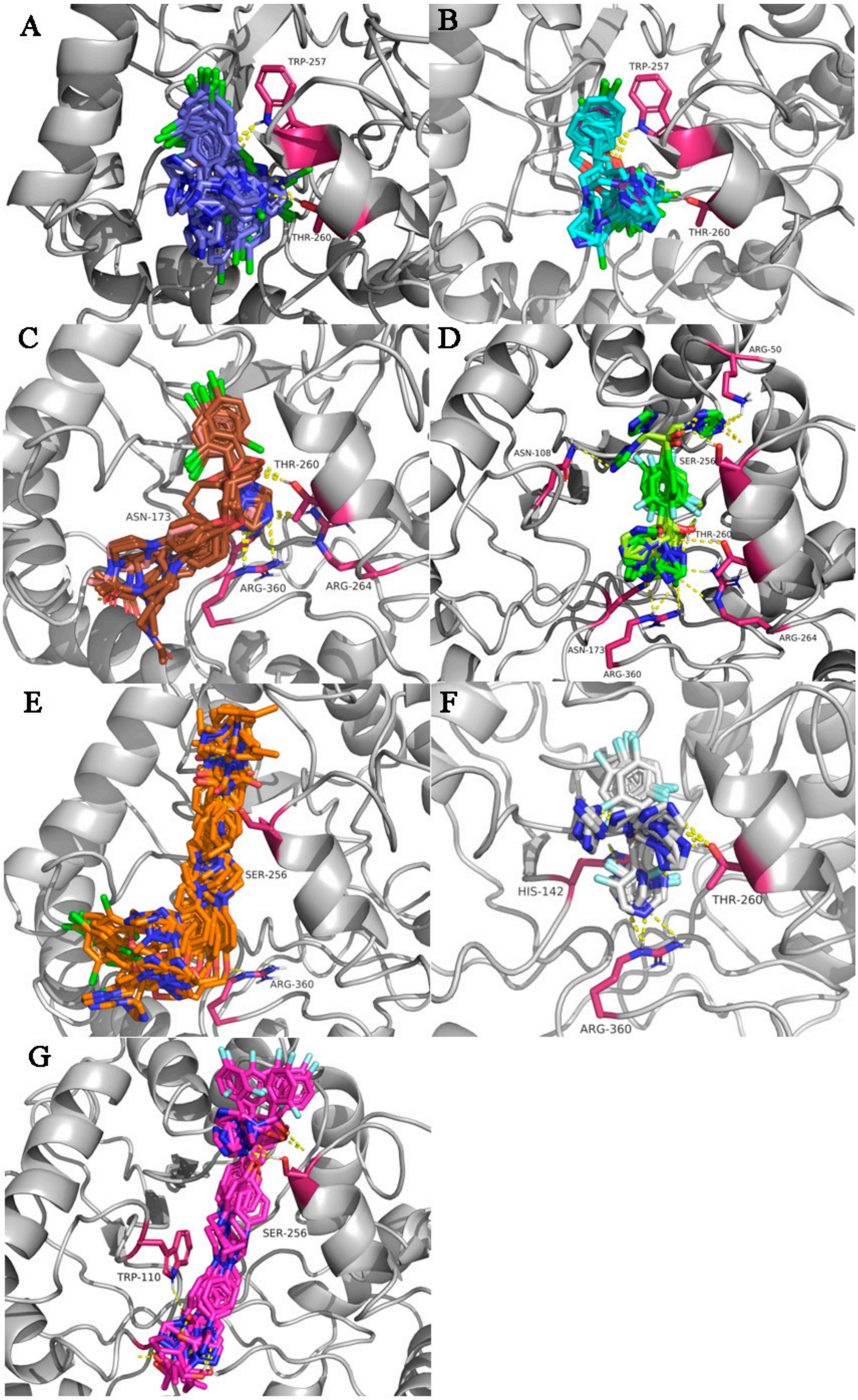


**Figure S10** The hydrogen bonds between the azoles and the residues in AKR7A2. A. Miconazole, B. Econazole, C. Ketoconazole, D. Fluconazole, E. Itraconazole, F. Voriconazole, G. Posaconazole

**Table S1.** Kinetic parameters of effect of voriconazole on AKR7A2 enzyme activity with SSA as a substrate

| Protein/drug | Drug concentration (μM) | $K_m$ (μM) | $k_{cat}$ ($min^{-1}$) | $k_{cat}/K_m$ ($min^{-1}*\mu M^{-1}$) | Normalized Value ($k_{cat}/K_m$) |
|---|---|---|---|---|---|
| AKR7A2 | 0 | 22.65±1.23 | 74.98±1.48 | 3.31 | 1.00 |
| voriconazole | 5 | 20.44±1.63 | 77.83±2.26 | 3.81* | 1.15 |
| voriconazole | 20 | 18.99±1.02 | 78.36±1.44 | 4.13** | 1.25 |
| voriconazole | 50 | 17.30±0.88 | 79.54±1.33 | 4.60*** | 1.39 |

$^{*}p < 0.05$, $^{**}p < 0.01$, $^{***}p < 0.001$. The data represents the mean ±SD of at least three replicates and were analyzed by GraphPad Prism 8.0.
